# Characterization of Blf4, an Archaeal Lytic Virus Targeting a Member of the Methanomicrobiales

**DOI:** 10.3390/v13101934

**Published:** 2021-09-26

**Authors:** Katrin Weidenbach, Sandro Wolf, Anne Kupczok, Tobias Kern, Martin A. Fischer, Jochen Reetz, Natalia Urbańska, Sven Künzel, Ruth A. Schmitz, Michael Rother

**Affiliations:** 1 Institute for General Microbiology, Christian-Albrechts-Universität zu Kiel, 24118 Kiel, Germany; kweidenbach@ifam.uni-kiel.de (K.W.); anne.kupczok@wur.nl (A.K.); FischerM@rki.de (M.A.F.); rschmitz@ifam.uni-kiel.de (R.A.S.); 2 Institute of Microbiology, Technische Universität Dresden, 01062 Dresden, Germany; sandro.wolf@gmx.de (S.W.); tobskern@googlemail.com (T.K.); nataliaurbanska.9595@gmail.com (N.U.); 3 Bioinformatics Group, Plant Sciences Group, Wageningen University & Research, 6708 PE Wageningen, The Netherlands; 4 German Federal Institute for Risk Assessment, 10589 Berlin, Germany; reetz1952@googlemail.com; 5 Max-Planck-Institute for Evolutionary Biology, 24306 Plön, Germany; kuenzel@evolbio.mpg.de

**Keywords:** archaea, biogas, *Methanoculleus* sp., virus, *Siphoviridae*, genome sequence

## Abstract

Today, the number of known viruses infecting methanogenic archaea is limited. Here, we report on a novel lytic virus, designated Blf4, and its host strain *Methanoculleus bourgensis* E02.3, a methanogenic archaeon belonging to the Methanomicrobiales, both isolated from a commercial biogas plant in Germany. The virus consists of an icosahedral head 60 nm in diameter and a long non-contractile tail of 125 nm in length, which is consistent with the new isolate belonging to the *Siphoviridae* family. Electron microscopy revealed that Blf4 attaches to the vegetative cells of *M. bourgensis* E02.3 as well as to cellular appendages. Apart from *M. bourgensis* E02.3, none of the tested *Methanoculleus* strains were lysed by Blf4, indicating a narrow host range. The complete 37 kb dsDNA genome of Blf4 contains 63 open reading frames (ORFs), all organized in the same transcriptional direction. For most of the ORFs, potential functions were predicted. In addition, the genome of the host *M. bourgensis* E02.3 was sequenced and assembled, resulting in a 2.6 Mbp draft genome consisting of nine contigs. All genes required for a hydrogenotrophic lifestyle were predicted. A CRISPR/Cas system (type I-U) was identified with six spacers directed against Blf4, indicating that this defense system might not be very efficient in fending off invading Blf4 virus.

## 1. Introduction

Methanogenesis, biogenic methane formation, is a metabolic trait found exclusively in certain members of the Archaea. These methanogenic archaea (methanogens) thrive by coupling the conversion of simple C1 and C2 compounds (such as CO_2_, formate, methanol, or acetate) to methane with energy conservation [1,2]. This unique metabolic capacity makes methanogens highly relevant in mitigating climate change. On the one hand, methane is a potent greenhouse gas that contributes significantly to global warming [3]. On the other hand, methane is a clean energy source, as its combustion with oxygen produces only CO_2_ and water [4]. One strategy to provide regenerative methane (biogas) in times of dwindling fossil fuels is through anaerobic digestion. This multi-step process, which leads to the decomposition of biomass or waste, involves a plethora of different anaerobic microorganisms, with methanogens operating at the terminal step [5]. Cultivated methanogens are currently classified into seven orders, Methanococcales, Methanopyrales, Methanocellales, Methanobacteriales, Methanomassiliicoccales, Methanosarcinales, and Methanomicrobiales [6]. Of the latter two, the genera *Methanosarcina* and *Methanoculleus* are particularly abundant in biogas plants fed with energy crops [7,8], which indicates their crucial role in the process.

In all types of ecosystems, viruses play an important role in affecting the abundance of microorganisms, the composition of microbial communities, and the dynamics of microbial populations [9,10,11]. There are currently 17 archaeal virus families described, which are divided into archaea-specific and cosmopolitan virus families based on their morphology and genome characteristics. The archaea-specific virus families are often characterized by their unique morphology (for reviews see, e.g., [12,13,14]). The cosmopolitan group includes the Caudovirales, which are divided into the *Podoviridae*, the *Siphoviridae*, the *Myoviridae*, and the Magroviruses [14]. In addition to the previously known archaeal virus families, the analysis of metagenome data offers a large repertory for the discovery of new archaeal viruses and virus families.

In addition to defense mechanisms against foreign nucleic acids, such as restriction–modification (RM) systems, prokaryotes often have clustered regularly interspaced palindromic repeats (CRISPR)/CRISPR-associated (Cas) systems, which are the adaptive prokaryotic immunity. They are present in most sequenced archaeal genomes (>90%). Today, the CRISPR/Cas systems are divided into 2 classes, 6 types, and >30 subtypes [15,16]. The system consists of a CRISPR array and genes for Cas proteins. The array is organized into spacer sequences, which are short pieces of DNA from previous invaders (20–40 bp) and repeats (20–40 bp) that are positioned between the individual spacers. At the 5′-end of the array is the so-called leader sequence. In the event of an infection with foreign nucleic acids, the CRISPR/Cas system defends in three phases. In the adaptation phase, new spacers are added to the CRISPR array by the enzyme complex containing Cas 1 next to the leader sequence, whereby foreign DNA will later be recognized using the protospacer adjacent motif (PAM) sequence. In the expression phase, the array is transcribed and the precursor CRISPR RNA (pre-crRNA) is processed, which leads to short mature crRNAs. In the interference phase, the crRNA forms the interference complex with other Cas proteins, which degrades the foreign nucleic acids (for reviews see, e.g., [17,18]).

Whether CRISPR/Cas systems play a significant role in fending off viral infections of methanogenic archaea involved in anaerobic digestion is unclear. The emerging evidence for the relevance of viruses to the structure and population dynamics of archaeal communities is still largely based on observations *in situ* [19,20,21]. Most isolated and described viruses infecting methanogens are restricted to the Methanobacteriales, including the related *Methanothermobacter marburgensis*-infecting viruses ψM1 and ψM2, and the *Methanothermobacter wolfeii* provirus ψM100 [22,23,24]. Further examples are the viruses ΦF1, ΦF3, and Drs3, which infect members of the genus *Methanobacterium* [25,26]. For a member of the Methanococcales, *Methanococcus voltae*, virus(-like) particles were reported but not characterized in detail [27,28]. Recently, the first virus infecting a member of the Methanosarcinales (MetSV) was isolated and characterized [29]. A second virus infecting *Methanosarcina* sp. (MetMV) was predicted *via* a metagenomic approach [30]. For the other methanogenic orders, no virus has been described. From virus (phage) research on bacteria, as well as from isolation-independent approaches, such as metagenomics and genome-based searches for proviruses, it is very likely that virus diversity in methanogens, and the whole archaeal domain, is greatly under-appreciated [13,31].

In this study, we isolated *Methanoculleus bourgensis* E02.3 and its lytic virus, Blf4, from a commercial biogas plant in Germany. The virus belongs to the *Siphoviridae*, and is the first one described to infect a member of the Methanomicrobiales, expanding the repertoire of isolated viruses infecting methanogens.

## 2. Materials and Methods

### 2.1. Isolation and Characterization of Methanoculleus bourgensis E02.3

All manipulations were made under anaerobic conditions, either by applying standard anaerobic techniques [32] or by working in a glove box (Coy, Grass Lake, MI USA) operated with an atmosphere of N_2_/CO_2_/H_2_ (78:18:4, *v*/*v*). “Modified Basal Medium” (MBM) and solid “Modified Basal Agar” (MBA) were used to isolate and culture methanogenic archaea present in sludge obtained in April 2013 from a full-scale commercial anaerobic digester in Germany (designated “BG1” in [33]). MBM and MBA were prepared as described [26], without adding yeast extract and supplementing with sodium acetate (Carl Roth, Karlsruhe, Germany) to 40 mM. The sludge was serially diluted ten-fold, dispensed onto MBA plates, and incubated at 45 °C with H_2_/CO_2_ (80:20 (*v*/*v*), 1.5 × 10^5^ Pa) for 12 days. In this initial isolation step, the media contained 100 µg mL^−1^ (final concentration) of streptomycin and ampicillin each in order to inhibit bacterial growth. Obtaining a pure culture from a single colony on MBA and assessment of purity by phase-contrast and epifluorescence microscopy was conducted as described [26]. Growth was monitored photometrically by determining the optical density at 578 nm (OD_578_) or 600 nm (OD_600_) at 37 °C, 40 °C, 45 °C, and 50 °C. Growth onsodium formate (Merck, Darmstadt, Germany) at 45 °C was assessed as described [34]. Methanol for the growth experiments was from VWR (Dresden, Germany). Lysis of growing cultures by the virus was assessed by following their optical density after it was added.

The taxonomic rank of *M. bourgensis* E02.3, as well as its phylogeny, was assessed by analyzing a nearly complete fragment (1395 bp) of the 16S rRNA gene sequence, amplified by PCR. A phylogenetic tree was constructed using the neighbor-joining method and the Jukes–Cantor distance correction with MEGA based on a Geneious10.2.5 CLUSTAL W alignment of sequences representing the genus [35] and bootstrap values based on 1000 replications [36]. Additionally, the genome of *M. bourgensis* E02.3 was sequenced. Chromosomal DNA was isolated from *M. bourgensis* E02.3 using the Wizard Genomic DNA Purification Kit (Promega, Madison, WI, USA) as recommended by the manufacturer. Illumina shotgun paired-end sequencing libraries were prepared using the Nextera DNA Flex Library Preparation Kit (Illumina, San Diego, CA, USA) according to the manufacturer’s instructions. The sample was sequenced on the NextSeq500 using the NextSeq 500/550 Mid Output Kit v2.5 sequencing chemistry (300 cycles). Additionally, 400 ng of high-molecular-weight genomic DNA were sequenced using the SQK-RBK004 rapid sequencing kit, FLO-MINI106 flow cells, and a MinIon device (MinKNOWversion 20.06.4) (Oxford Nanopore Technologies, Oxford, United Kingdom). Of the 2,213,275 paired-end reads, 15% overlapped and were merged using bbmerge [37]. A hybrid assembly of the merged and unmerged Illumina reads and MinION reads was performed using SPAdes v3.13 [38], and open reading frames (ORFs) were automatically annotated [39]. Read coverage was determined using the samtools depth function after mapping the Illumina data with BWA-MEM [40] and the MinIon data with Vulcan [41], respectively. Manual curation was performed to identify the genes for methanogenesis, archaeal flagellum, restriction modification systems (BLASTN; https://blast.ncbi.nlm.nih.gov/Blast.cgi; accessed on 8 March 2021); CRISPR/Cas systems were identified using CRISPRFinder [42], MacSyFinder [43], and CRISPRCasFinder [44]) and emboss_needle for the alignment of the *fla*-genes [45]. Protospacers were predicted from a CRISPR array in order to manually derive PAM motifs at the 5’ end of the Blf4 genome leading strand, and design logos using Weblogo software [46,47]. The draft genome sequence of *M. bourgensis* E02.3 is available at GenBank under the accession number GCA_018495055.1.

### 2.2. Isolation and Characterization of Blf4 Virus 

Sludge from the anaerobic digester was diluted 1:4 with MBM and manually homogenized. After centrifugation at 3000× *g* for 10 min, the supernatant was filtered through a 0.45 µm membrane. *M. bourgensis* E02.3 was infected with the filtrate in a standard double-layer plaque assay analogous to a procedure described previously [26]. Briefly, 1 mL of a serial 10-fold filtrate dilution (in MBM) was mixed with 1 mL of exponentially (OD_578_ of approximately 0.1) growing strain E02.3, combined with 2.5 mL of semi-solid molten MBA (0.7% (w/v) agar), and poured on fresh MBA plates. The plates were incubated at 45 °C with H_2_/CO_2_ for 14 days. Single plaques were harvested, serially diluted in MBM, and the lytic virus was again isolated *via* the plaque assays to ensure that a pure strain was obtained. For amplification of virus biomass, the volumes of the Blf4 lysate (up to 8 mL) were increased to lyse increasing volumes (up to 160 mL) of *M. bourgensis* E02.3 cultures incubated with slight agitation at 45 °C over several infection/lysis cycles. For subsequent analyses, the cultures were cleared by centrifugation at 10,000× *g* for 30 min after complete cell lysis (24–48 h) had occurred, and aliquots of the supernatants were either stored anaerobically in 5% (*v*/*v*) glycerol at −80 °C (Blf4 virus stock), subjected to isolation of the virus (see below), or used to assess the host spectrum of the Blf4 virus. For the latter, three *Methanoculleus* species, *M. bourgensis* MS2^T^ (DSM 3045, type strain), *M. marisnigri* AN8 (DSM 4552), and *M. thermophilus* CR1^T^ (DSM 2373, type strain) (Deutsche Sammlung für Mikroorganismen und Zellkulturen, DSMZ, Braunschweig, Germany), were cultivated in the respective media suggested by DSMZ (DSM 3045 in medium 332 at 37 °C, DSM 4552 in medium 141b at 37 °C, and DSM 2373 in medium 141 with strain-specific modifications at 55 °C) with H_2_/CO_2_ (80:20) and supplemented daily, as the energy substrate. For lysis assays, strains were grown in 5 or 50 mL culture to an optical density of 0.15–0.2 and supplemented with 0.2–0.5 mL of virus lysate passed through a 0.2 µm sterile filter (Sarstedt, Nümbrecht, Germany). The optical density was followed until complete lysis occurred or the stationary phase was reached.

The Blf4 virus was visualized by transmission electron microscopy (TEM) using conventional negative staining. To this end, virus stock suspension or Blf4-infected *M. bourgensis* E02.3 was applied to either pioloform- or formvar-carbon-coated, 300- or 400-mesh copper grids (Plano GmbH, Wetzlar, Germany) stained with 1–2% aqueous uranyl acetate solution and examined by TEM (JEM-1010, JEOL, Tokyo, Japan or Morgagni 268D, Thermo Fisher Scientific, Waltham, MA, USA) at an accelerated voltage of 80 kV.

### 2.3. Genomic Analysis of Blf4 Virus 

Lysate (80 mL) containing Blf4 virus was thoroughly mixed with chloroform at a final concentration of 5% (*v*/*v*), incubated at 4 °C for 16 h, and centrifuged at 3000× *g* for 20 min. The supernatant was passed through a 0.45 µm pore filter (Filtropur S 0.45, Sarstedt, Germany) and subjected to ultracentrifugation (120,000× *g*, 2 h, 4 °C). The sediment was resuspended and, after another ultracentrifugation under the same condition, further purified by ultrafiltration through a 100 kDa molecular weight cut-off microcentrifuge device (Pall Corporation, Port Washington, NY, USA) at 1000× *g*. The retentate was treated with DNaseI (Thermo Fisher Scientific) to remove host DNA.

DNA was extracted from the isolated virus using the High Pure Viral Nucleic Acid kit (Roche, Mannheim, Germany) as per the manufacturer’s instructions. Illumina short-read shotgun sequencing of virus DNA from Blf4 with standard NGS library preparation, followed by paired-end 300 bp sequencing on a MiSeq system (Illumina) was conducted at the Genome Center of the Technische Universität Dresden. The sequencing run generated 7,813,662 paired-end raw reads. Filtering and subsampling were conducted as described [26]. Assembly was done using SPAdes v3.11.1 [48] with the “plasmid” and “careful” options selected. The resulting contigs were processed by Recycler to yield one circular contig [49]. The presence of a circular contig was further supported by cutting the contig *in silico* at an intergenic region and joining the ends to a novel contig. The resulting read mapping with BWA-MEM was contiguous and did not support any break point [40]. Annotation was done using RAST [50]. Further functions were assigned using BLASTP (NCBI, Bethesda MD, USA), phyre2 [51], and the antiCRISPR protein prediction tool PaCRISPR [52]. Virus classification was performed using VIRFAM by searching for the head–neck–tail module and recombinase [53,54]. The genome sequence of Blf4 is available at NCBI under the accession number MZ171369.

## 3. Results

### 3.1. Initial Characterization of M. bourgensis E02.3

**Figure 1:**
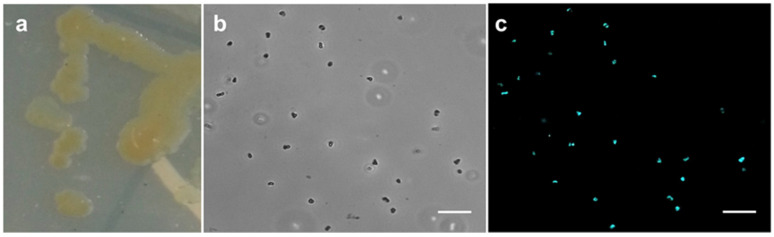
Morphology of methanogenic isolate E02.3. Colonies on MBA (**a**), cells visualized by phase contrast (**b**), and fluorescence microscopy (**c**); scale bars = 10 μm.

**Figure 2:**
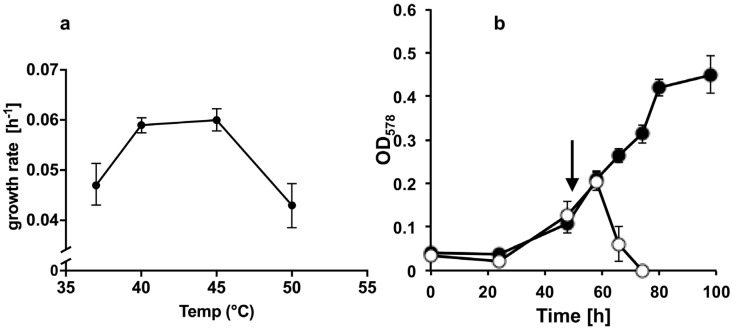
Growth rate of *M. bourgensis* E02.3 dependent on temperature (**a**) and lysis of by Blf4 virus at 45 °C (**b**); growth of *M. bourgensis* E02.3 was monitored at 578 nm over time and the growth rate (h^−1^) was calculated for the exponential phase. Filled circles: untreated cultures; open circles: cultures challenged with Blf4 after 48 h (arrow). To save space, the y-axis of (**a**) is shown discontinuously. The average values and their standard deviations (error bars) of three biological replicates are shown.

The methanogenic strain E02.3 was isolated from the sludge of a commercial biogas plant in Germany (see Section 2.1). E02.3 formed round, yellow to greenish, shiny colonies of approximately 5 mm in diameter on MBA (Figure 1a). The cells were coccoid with a diameter of 0.5–2 µm (Figure 1b) and exhibited F_420_ autofluorescence (Figure 1c), which is a hallmark of methanogens. During inspection by light microscopy, motility of the E02.3 strain was not observed. The strictly anaerobic strain was able to utilize H_2_/CO_2_ (80:20, *v*/*v*; 1.5 × 10^5^ Pa) or formate (150 mM) for growth. Growth with methanol (125 mM) or acetate (120 mM) as sole energy sources was not observed. Growth of the E02.3 strain required acetate (10–40 mM gave no phenotypic difference) to be present in the medium. Growth was fastest at 40 °C and 45 °C (Figure 2a), the temperature range at which the biogas plant was operated. Growth was apparently impaired at 37 °C and 50 °C (Figure 2a).

Comparative analysis of the nearly complete 16S rRNA gene (1395 bp) from the E02.3 isolate with other sequences in GenBank (BLASTN) showed that this strain belongs to the genus *Methanoculleus*, being most closely related to *M. bourgensis* CB-1 (nucleotide identity of 99.9%, accession number AB065298) (Supplementary Figure S1). Both acetate auxotrophy and the doubling time of approximately 12 h correspond to *M. bourgensis*’ phenotype [55] and are, thus, consistent with this conclusion. For further analysis, chromosomal DNA was isolated and sequenced using Illumina and Oxford Nanopore technology. We obtained 7,813,662 paired-end Illumina reads of lengths of 300 bp and 58,777 Minion reads with lengths between 103 bp and 62,913 bp (average 3053 bp). The draft genome with nine contigs (total length of 2,643,145 bp) was well covered by Illumina reads (average coverage of 239, standard deviation of 51) and Minion reads (average coverage of 63, standard deviation of 10), and had an average GC content of around 61% (Supplementary Table S1). The calculated genome size of approximately 2.6 Mbp is similar to those of the *M. bourgensis* strains MAB1 and MS2^T^ (Supplementary Table S2). Based on automated annotation, most enzymes for hydrogenotrophic methanogenesis were encoded on contig 1 and localized in a large cluster (nt 460089–479419), which is highly similar to a cluster in *M. bourgensis* strains MS2^T^ and MAB1. In order to identify the genes for the proteins that are necessary for growth on other methanogenic substrates, a search was performed for the corresponding genes in the strains *M. bourgensis* MS2^T^ and MAB1 (present in the NCBI database) and a BLASTN analysis of the *M. bourgensis* E02.3 draft genome. The genes encoding formate dehydrogenase (required for growth on formate; BN140_1327/_1328/_1329) were found in contig 4. Genes for (potentially redundant) enzymes involved in the assimilation of acetate were found scattered across the genome—putative acetate-CoA ligase (BN140_034) in contig 2, acetate kinase (BN140_1312) in contig 4, phosphotransacetylase (BN140_0884) in contig 3, acetyl-CoA-synthetase (BN140_2190) in contigs 8 and 5, and carbon monoxide dehydrogenase/acetyl-CoA synthase complex (MMAB1_3162) in contig 4.

**Figure 3:**
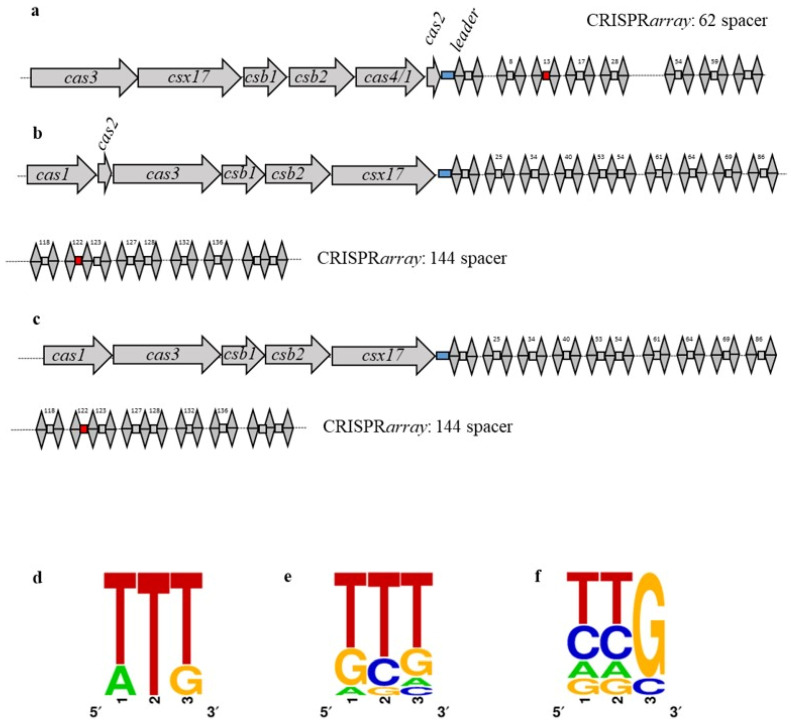
CRISPR/Cas system type I-U of *M. bourgensis* E02.3 and predicted PAM motifs: (**a**) CRISPR/Cas system in *M. bourgensis* E02.3 contig 7. It consists of the genes for Cas3, Csx17, Csb1, Csb2- and Cas4/1-fusion, and Cas2. The array includes 63 directed repeats with a length of 36 nt, which were arranged alternately with spacers. (**b**) CRISPR/Cas system in *M. bourgensis* MAB1 type I-U_I, and (**c**) CRISPR/Cas system in *M. bourgensis* MS2^T^ type I-U_I. They consist of the genes for the Cas proteins Cas1, (only *M. bourgensis* MAB1, Cas 2), Cas 3, Csb1, Csb2, and Csx 17. The arrays are identical, including 144 spacers and the same direct repeats as the *M. bourgensis* E02.3 type I-U system. PAM motifs were predicted by manual curation of the Blf4 specific spacers for the strains (**d**) *M. bourgensis* E02.3, (**e**) *M. bourgensis* MS2^T^, and (**f**) *M. bourgensis* MAB1. Only one common spacer against Blf4 was identified (indicated in red). The leader sequence is marked in blue.

One locus encoding the proteins for the archaeal flagellum was identified (*flaJ*, *flaI*, *flaH*, *flaF*, *flaG*, and the flagellin *flaB*) in contig 1. The deduced proteins were 48.1 to 89.5% identical to those from *M. thermophilus* and *M. marisnigri*. A CRISPR/Cas system type I-U was identified in contig 7 (see Section 2.1). It consists of an array with 63 direct repeats (62 spacers of 36 to 41 nt in length) and genes for Cas3, Csx17, Csb1, Csb2, Cas4/1-fusion, and Cas2 protein (Figure 3a). *M. bourgensis* strains MAB1 (Figure 3b) and MS2^T^ (Figure 3c) also encode a CRISPR/Cas system type I-U, but subtype I (I-U_I) [44]. In addition to a different arrangement of the corresponding genes for the Cas proteins, the identical array of *M. bourgensis* MAB1 and MS2^T^ also contains more spacers (144) (Figure 3a–c). The repeat unit of all three *M. bourgensis* strains, E02.3, MS2^T^, and MAB1, was identical (36 nt).

Specific spacers against the Blf4 virus were identified by a direct search of the spacer sequences against the Blf4 genome (accession number MZ171369) using BLASTN. Thus, spacers with mismatches were also analyzed. Six spacers against the Blf4 virus were identified in the *M. bourgensis* E02.3 array, in which only two spacers had one mismatch (spacers 13 and 17), three spacers had two mismatches (spacers 8, 54, and 59), and one had five mismatches (spacer 28). In comparison, *M. bourgensis* MS2^T^ and MAB1 had 16 spacers against the Blf4 virus (two without mismatches). No spacers without a mismatch were present in the array of *M. bourgensis* E02.3 and only two spacers completely identical to Blf4 were present in the arrays of *M. bourgensis* MAB1 and MS2^T^, respectively. It has been shown that a 100% match is not necessary for the functionality of CRISPR/Cas systems [56,57]. Notably, strains E02.3, MS2^T^, and MAB1 had only one spacer in common (E02.3 spacer 13 and MS2^T^/MAB1 spacer 122; two mismatches to Blf4—red boxes in Figure 3) the other Blf4-specific spacers were completely different. Using the six spacers from *M. bourgensis* E02.3, a PAM motif was predicted (Figure 3d) using the 5´end of the Blf4 genome leading strand. Highly similar PAM motifs for Blf4 were also derived from *M. bourgensis* MS2^T^ and MAB1 (Figure 3e,f).

As for further mechanisms of defense against foreign DNA, restriction–modification systems were searched in the genome sequence of *M. bourgensis* E02.3. The draft genome was compared with the known restriction–modification systems from *M. bourgensis* MS2^T^ and MAB1 (using BLASTN). Genes were identified, which are annotated in *M. bourgensis* MS2^T^ and MAB1 as enzymes of the type III restriction–modification system in contig 3 (nt: 259043–263631). A further section in contig 3 is very similar to another type III restriction–modification of *M. bourgensis* MS2^T^ (BN140_0641/0642) and MAB1 (MMAB1_0829/0830), but an intermediate section of 1251 bp (contig 3; 5255–6470 nt) differs significantly in the primary structure. Surprisingly, none of the known type I restriction–modification systems from *M. bourgensis* MS2^T^ (BN140_0921-0923; BN140_1098-1100) or MAB1 (MMAB1_2046-2048) were identified.

### 3.2. Characterization of the Blf4 Virus 

**Figure 4:**
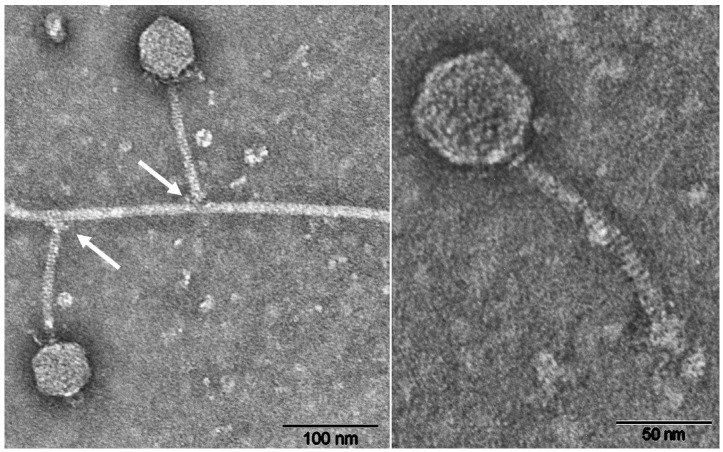
TEM micrographs of the Blf4 virus. Left: virus attachment to cell appendage (arrows), presumably flagellum. Right: a single virus displaying typical morphological characteristics of members of the *Siphoviridae*; Blf4 contains a hexagonal head (approximately 60 nm in diameter) and a non-contractile tail (approximately 125 nm in length and 10 nm wide). The samples were negatively stained with uranyl acetate (bars = 100 nm and 50 nm in the left and right images, respectively).

Blf4 completely lysed cultures of *M. bourgensis* E02.3 24 h after they were challenged with the virus (Figure 2b). TEM analysis of purified Blf4 suggested its affiliation with the virus family *Siphoviridae*. The non-contractile tail was approximately 125 nm in length and 10 nm in width. It was straight or curved and had a slightly enlarged terminal segment. The nearly isometric hexagonal head was approximately 60 nm in diameter (Figure 4). When Blf4-infected *M. bourgensis* E02.3 was analyzed by TEM, the virus was seen attached to vegetative cells, and, less frequently, to cellular appendages of E02.3, which are presumably archaeal flagella (Supplementary Figure S2). Flagellotropy (i.e., attaching to flagella) of members of the *Siphoviridae* is not uncommon [58,59].

The host range of Blf4 was investigated by challenging various *Methanoculleus* strains (*M. bourgensis* MS2^T^, *M. marisnigri* AN8, and *M. thermophilus* CR1^T^; see Section 2.2) in liquid culture with the virus. For none of these strains could cell lysis be observed within 72 h after infecting exponentially growing hosts (Supplementary Figure S3).

The assembly of sequencing reads generated one high coverage contig of 37,078 bp in length (average coverage of 876, standard deviation of 105). This length is comparable to other members of the *Siphoviridae*-infecting archaeal hosts (from approximately 26 kbp for ψM2 up to approximately 42 kbp for BJ1) [14]. The GC content of 63.1% is close to the calculated GC content of its host (60–62%, see Supplementary Table S1). While the read data support the notion that the genome is circular, no terminal repeats or similar indicators were found, and no physical experiments were conducted in this regard. Thus, beyond bioinformatic indications, no further evidence is present to substantiate this notion. Of the 63 ORFs identified within Blf4, manual analysis (see Section 2.3) allowed for assigning functions to most of them (Supplementary Table S3). All ORFs were transcriptionally organized in the same direction. The virus genome is structured as follows: ORF 1 and 2 encode putative membrane proteins and ORF 7 encodes a phage terminase. ORFs 10 and 11 encode phage portal proteins commonly found in members of the Caudovirales, which also supports classifying Blf4 to the *Siphoviridae*. The portal proteins are involved in virus replication (virion assembly), DNA packing, and DNA delivery [60]. ORFs 12 to 34 encode its structural components, such as tail and capsid. This genomic region is followed by ORFs encoding functions necessary for the interaction with the host (e.g., (*S*-adenosyl-dependent) methyltransferases, potential antiCRISPR proteins), and for the maturation of the virus (e.g., HNH domain-containing protein) and the products of ORFs 62 and 63 are putative endonucleases. Further genome analysis using VIRFAM for a head–neck–tail module search supported the classification as *Siphoviridae* type 1 (no cluster assigned), as well as protein function prediction for the major capsid protein (MCP)(ORF14), the portal protein (ORF11), the terminase (ORF7), the head–tail adapter protein (ORF16), the head closure protein (ORF17), the neck protein (ORF19), and the tail completion protein (ORF20) (see Supplementary Tables S3 and S4). No significant similarities were detected using BLASTP for the other four *Siphoviridae* type 1 infecting archaea (archaeal virus BJ1 (taxid 416419), *Methanothermobacter wolfeii* prophage ψM100 (taxid 173824), *Methanobacterium* phage ψM2 (taxid 77048), *Methanobacterium formicicum* virus Drs3 (taxid 1430441), *Methanosarcina*-infecting virus MetSV (taxid 2035535), and *Halorubrum* phage CGphi46 (taxid 75406). Furthermore, VIRFAM (head–neck–tail module) analysis of these four *Siphoviridae* members infecting archaea and Blf4 showed, beside the different genome sizes, different arrangements of the genes for the structural proteins (Supplementary Figure S4).

## 4. Discussion

The Blf4 virus described here is only one of the few known to infect methanogenic archaea. Blf4 virus and its host, *M. bourgensis* E02.3, were isolated from a commercial biogas plant operated at approximately 40 °C [33]. The growth temperature range (Figure 2a) is consistent with this environment. Phylogenetic analysis suggests that strain E02.3 is a member of the genus *Methanoculleus*. The high degree of protein similarity that was deduced from the genomic sequence to other *M. bourgensis* strains (MS2^T^ or MAB1), for example, those involved in energy metabolism (hydrogenotrophic methanogenesis), supports this notion.

The Blf4 virus consists of an isometric icosahedral head and a non-contractile tail, which suggests that it belongs to the *Siphoviridae* virus family. Viruses of this family are known to infect bacteria or archaea. The morphology of Blf4 resembles that of the methanogenic viruses Drs3, ψM1, and ΦF3 [23,25,26]. The genome of Blf4 might be organized in a circular manner. However, more common to *Siphoviridae* is a circular, permuted, terminally redundant genome that is packaged by a “headful mechanism” as linear dsDNA into the viral particle, and circularizing after injection into the host [60]. Potential functions were predicted for most of Blf4′s genes. The genome appears to be divided into three regions—a region encoding proteins involved in replication, a region encoding structural proteins, followed by a region encoding proteins involved in virus maturation and host–virus interaction. The arrangement of ORFs in these regions into functionally distinct groups could potentially result in temporally distinct transcription patterns representing early and late viral genes, as reported for other viruses [61,62,63,64,65]. According to our prediction of a portal protein, a phage terminase (large subunit), and an HNH nuclease, it is likely that the genome of Blf4 is packaged using a type II packaging system, as is known for other dsDNA-containing tailed viruses, such as the *Escherichia coli* phage HK97 (reviewed in [66]).

In addition to proteins for virus propagation and structure, Blf4 also codes for proteins that may be involved in host defense inhibition, namely antiCRISPR (Acr) proteins, which are able to interfere with the host’s CRISPR/Cas system defenses (reviewed in [67]). Only a few Acrs have been described and they mainly originate from viruses infecting *Pseudomonas*, *Pectobacterium*, *Listeria*, *Streptococcus*, *Moraxella*, or *Sulfolobus* [68,69,70,71,72,73]. Here, Acrs that have been predicted in the archaeal Blf4 virus share only low similarities to known Acrs. Still, they might have enabled Blf4 to infect *M. bourgensis* E02.3, despite the presence of a CRISPR/Cas system primed against it. The function of putative Blf4 Acrs and their potential interaction with the host’s CRISPR/Cas system is unclear, but it is expected to differ from the SIRV2 Acrs [67], as (a) the CRISPR/Cas systems of the hosts differ (*Sulfolobus* contains types I-A, I-D, and III-B; *M. bourgensis* E02.3 contains type I-U), and (b) no similarity between the respective Acrs could be detected. Possible explanations for the very narrow host range of Blf4 observed in this study might be the fact that *M. bourgensis* E02.3 lacks type I restriction–modification systems, which is encoded by other *M. bourgensis* (e.g., MS2^T^ or MAB1), or the presence of more and different spacers against the virus in the CRISPR array of the tested strains.

The finding that Blf4 attaches to both *M. bourgensis* E02.3′s cell surface and its appendages (Figure 4, Supplementary Figure S2) suggests that the virus might be facultatively flagellotropic. This would increase the target radius (up to 10-fold) and allow for movement of the virus from the flagella towards a second receptor on the cell surface [74,75]. Such facultative flagellotropy raises the question about the array of epitopes Blf4 recognizes. *M. thermophilus* and *M. marisnigri* contain flagella (particularly, FlaB), which are rather distinct from those of *M. bourgensis* E02.3. Both strains were not lysed by Blf4. In contrast, *M. bourgensis* MS2^T^, when challenged with Blf4, was also not lysed, despite encoding a flagellum very similar to that of *M. bourgensis* E02.3. The observed immunity against Blf4 in the *Methanoculleus* strains examined here might be based on a combination of different mechanisms, such as a defense system (CRISPR/Cas or a restriction–modification system in the case of *M. bourgensis* MS2^T^) and incompatible receptors (in the case of *M. thermophilus* or *M. marisnigri*).

Using culture-independent sequencing approaches, a number of methanogenic archaeal taxonomic groups have been identified as being potentially relevant for the biogas process. In biogas plants operated in a similar fashion to the one from which Blf4 originated, these abundant methanogens included the genera *Methanosarcina* and *Methanoculleus* [33]. In fact, members of the latter genus were repeatedly found to be the most abundant methanogens present in the biogas plant from which Blf4 was isolated [76]. Assuming that numerical abundance equals metabolic relevance and that each species is infected by at least one virus, Blf4 and similar ones infecting members of the *Methanoculleus* genus might exert a profound effect on their host’s abundance and, thus, on the efficiency and the economy of the whole biogas process. Since only a few viruses of methanogens are known, their overall impact on methanogenic activity can only be guessed. Therefore, exploring the genetic diversity of viruses infecting methanogens and the dynamics of virus–host interactions will aid our understanding of anaerobic digestion and of anaerobic systems in general.

## Data Availability

The data presented in this study are available in the main text or the Supplementary Material. Genome sequences are accessible at NCBI under accession numbers GCA_018495055.1 (*M. bourgensis* E02.3) and MZ171369 (Blf4 virus).

## Supplementary Materials

The following are available online at https://www.mdpi.com/article/10.3390/v13101934/s1. Figure S1: Phylogenetic position of strain E02.3 within the genus Methanoculleus; Figure S2: Attachment of virus Blf4 to M. bourgensis E02.3; Figure S3: Host range of virus Blf4; Figure S4: VIRFAM analysis by head–neck–tail module search; Table S1: GC content of the draft genome of M. bourgensis E02.3; Table S2: Genome sizes of M. bourgensis strains; Table S3: Open reading frames in the Blf4 genome with similarity to described proteins in databases.
