# Supplementary material for: Characterization of Blf4, an Archaeal Lytic Virus Targeting a Member of the Methanomicrobiales
Source: Viruses. 2021 Sep 26;13(10):1934. doi: 10.3390/v13101934 (PMC8540584; doi:10.3390/v13101934)
Supplement: Supplementary file 1 [file viruses-13-01934-s001.zip › viruses-1316505-supplementary.pdf]

Supplementary Information

Characterization of Blf4, an archaeal lytic virus targeting a member of the Methanomicrobiales

Katrin Weidenbach, Sandro Wolf, Anne Kupczok, Tobias Kern, Martin A. Fischer, Jochen Reetz, Natalia Urbańska, Sven Künzel, Ruth A. Schmitz and Michael Rother

Supplementary Table S1: GC content of the draft genome of *M. bourgensis* E02.3

contig number	contig length (bp)	GC content
contig 1	710727	60.7 %
contig 2	496511	60.8 %
contig 3	342169	60 %
contig 4	275614	60.8 %
contig 5	237631	59 %
contig 6	205485	61.3 %
contig 7	147952	62.1 %
contig 8	141118	61.7 %
contig 9	85938	62.2 %

Supplementary Table S2: Genome sizes of *M. bourgensis* strains.

Strain	Accession number	Genome size
M. bourgensis E02.3	GCA_018495055.1	2,643,145 bp
M. bourgensis MAB1	LT158599	2,859,299 bp
M bourgensis MS2T	HE964772	2,789,774 bp

Supplementary Table S3: Open reading frames in the Blf4 genome with similarity to described proteins in databases.

ORF #	Start (nt)	Stop (nt)	Length[a]	Predicted function	Predicted with
1	32	301	89	putative membrane protein	Phyre2<br>Confidence: 45.2 %<br>Identity: 48 %
2	298	549	83	putative membrane protein	Phyre2<br>Confidence: 32.8 %<br>Identity: 20 %
3	546	692	43	hypothetical protein	RAST
4	685	1245	186	HTH transcription regulator	BLASTP<br>E-value: 1^{-10}
5	1242	2486	414	DNA modification methylase (replication)	RAST
6	2532	2942	136	hypothetical protein (DUF523 domain)	Phyre2<br>Confidence: 100 %<br>Identity: 40 %
7	2961	4436	491	phage terminase, large subunit (replication)	RAST<br>VIRFAM; seq. identity 19 – 36 %[c]
8	4540	4848	102	hypothetical protein	RAST
9	4845	5093	82	hypothetical protein	RAST
10	5161	6798	535	phage portal protein (structural function)	BLASTP:<br>E-value: 1^{-84}
11	6795	7604	269	phage portal protein (structural function)	BLASTP:<br>E-value: 7^{-36}<br>VIRFAM; seq. identity 19 %[c]
12	7601	8695	364	putative GNAT family N-acetyltransferase	BLASTP:<br>E-value: 2^{-73}
13	8698	9075	125	hypothetical protein (DUF2190 containing protein)	Phyre2<br>Confidence: 25.8 %

					Identity: 27 %
14	9076	10263	395	Major capsid protein (structural function)	VIRFAM; seq. identity 19-23 % [c]
15	10290	10430	46	putative viral transmembrane protein (gp41) (envelope; structural function)	Phyre2 Confidence: 15 % Identity: 33 %
16	10427	10795	122	head tail adapter protein (structural function)	VIRFAM; seq. identity 24 % [c]
17	10795	11145	116	head closure protein protein (structural function)	VIRFAM; seq. identity 11-16 % [c]
18	11142	11393	83	putative antiCRISPR protein	PaCRISPR score [b]: 0.51
19	11390	11815	141	HK97gp10 family phage protein -neck protein (structural function)	BLASTP: E-value: 1^{-16} VIRFAM; seq. identity 30-31 % [c]
20	11816	12199	127	tail terminator protein; tail completion	Phyre2 Confidence: 100 % Identity: 15 % VIRFAM; seq. identity 24-25 % [c]
21	12204	13004	266	hypothetical protein	RAST
22	13007	13366	119	hypothetical protein	RAST
23	13459	13620	53	tail fiber protein S (tail, structural function)	Phyre2 Confidence: 12.8 % Identity: 45 %
24	13610	16072	820	phage tail tape measure protein, TP901 family (tail, structural function)	RAST
25	16069	16230	53	phage tail protein or putative Acr	PaCRISPR score [b]: 0.605
26	16233	16580	115	capsid assembly protein (structural function)	Phyre2 Confidence: 15 % Identity: 16 %
27	16581	18068	495	phage protein	RAST
28	18148	18324	58	putative antiCRISPR protein	PaCRISPR score [b]: 0.641
29	18328	18759	143	putative envelope glycoprotein (structural function)	Phyre2 Confidence: 51.3 % Identity: 41 %
30	18756	19094	112	base plate upper protein (structural function)	BLASTP: E-value: 5^{-51}
31	19186	20046	286	viral protein	Phyre2 Confidence: 100 % Identity: 17 %
32	20052	20297	81	hypothetical protein	RAST
33	20294	22471	725	Siphovirus Regi Pepy 6 GP37 family protein	BLASTP: E-value: 1^{-82}
34	22468	23640	390	Tail fiber protein (structural function)	BLASTP: E-value: 2^{-23}

35	23678	24022	114	hypothetical protein	RAST
36	24185	24550	121	putative antiCRISPR protein	PaCRISPR score [b]: 0.611
37	24761	24982	73	putative antiCRISPR protein	PaCRISPR score [b]: 0.769
38	25150	25350	66	hypothetical protein	RAST
39	25440	25607	55	hypothetical protein (fold: Ribbon-helix-helix)	Phyre2 Confidence: 35.5 % Identity: 39 %
40	25604	25948	114	putative antiCRISPR protein	PaCRISPR score [b]: 0.535
41	25933	26304	123	putative antiCRISPR protein	PaCRISPR score [b]: 0.605
42	26304	26486	60	BC1881 family protein	BLASTP: evalue: 6e-04
43	26486	26905	139	putative antiCRISPR protein	PaCRISPR score [b]: 0.589
44	26902	27201	99	putative antiCRISPR protein	PaCRISPR score [b]: 0.519
45	27201	27359	52	RecT family protein (recombination)	BLASTP: E-value: 3^{-11}
46	27356	28051	231	hypothetical protein	RAST
47	28051	28311	86	hypothetical protein Fold: DNA/RNA 3 helical bundle	Phyre2 Confidence: 18.8 % Identity: 27 %
48	28308	28736	142	putative antiCRISPR protein	PaCRISPR score [b]: 0.644
49	28741	29109	122	HNH endonuclease/recombinase	BLASTP: E-value: 3^{-04}
50	29106	29294	62	capsid	Phyre2 Confidence: 12 % Identity: 44 %
51	29291	29539	82	hypothetical protein	RAST
52	29546	30763	405	plasmid replication/partition related protein (replication)	RAST
53	30753	31322	189	hypothetical protein	RAST
54	31319	31696	125	hypothetical protein	RAST
55	31734	32126	130	Isomerase; DNA-binding protein:gyrase (replication)	Phyre2 Confidence: 61.6 % Identity: 29 %
56	32123	32263	46	SAM-Mtase	Phyre2 Confidence: 11 % Identity: 28 %
57	32363	32560	65	Hypothetical protein	RAST
58	32557	33750	397	Adenine-specific methyltransferase (EC 2.1.1.72)	RAST
59	33747	35816	689	DNA primase/helicase, phage-associated	RAST

60	35782	36111	109	hypothetical protein	RAST
61	36112	36429	105	hypothetical protein	RAST
62	36426	36911	161	putative hydrolase/restrictions endonuclease	Phyre2<br>Confidence: 93.4 %<br>Identity: 20 %
63	36911	37078	55	exonuclease	Phyre2<br>Confidence: 8.1 %<br>Identity: 14 %

a: number of amino acids
b: probability to be an antiCRSPR protein (https://pacrispr.erc.monash.edu)
c: highest identities of deduced Blf4 proteins with at least three proteins in Aclame phages (http://aclame.ulb.ac.be)

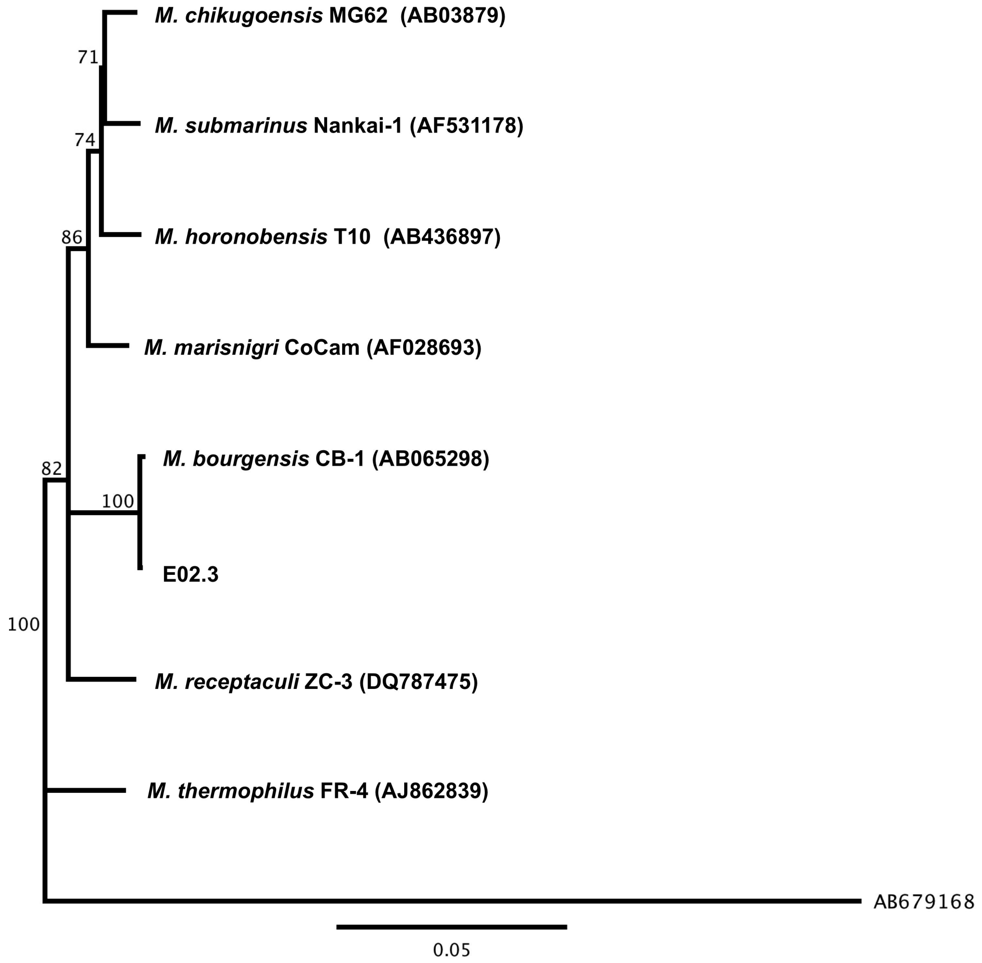

Supplementary Figure S1: Phylogenetic position of strain E02.3 within the genus *Methanoculleus*. Neighbor-joining tree constructed using MEGA in Geneious10.2.5 from a CLUSTAL W alignment of a 16S rRNA gene fragments (1,395 bp) of strain E02.3 and of corresponding sequences representing the genus *Methanoculleus* (GenBank accession numbers in parentheses), with *Methanotrix concilii* NBRC 103675 (AB679168) as the outgroup. Distance was corrected via Jukes & Cantor, the bootstrap values are based on 1000 replications; only values >50 % are shown. Bar, 0.05 evolutionary distance.

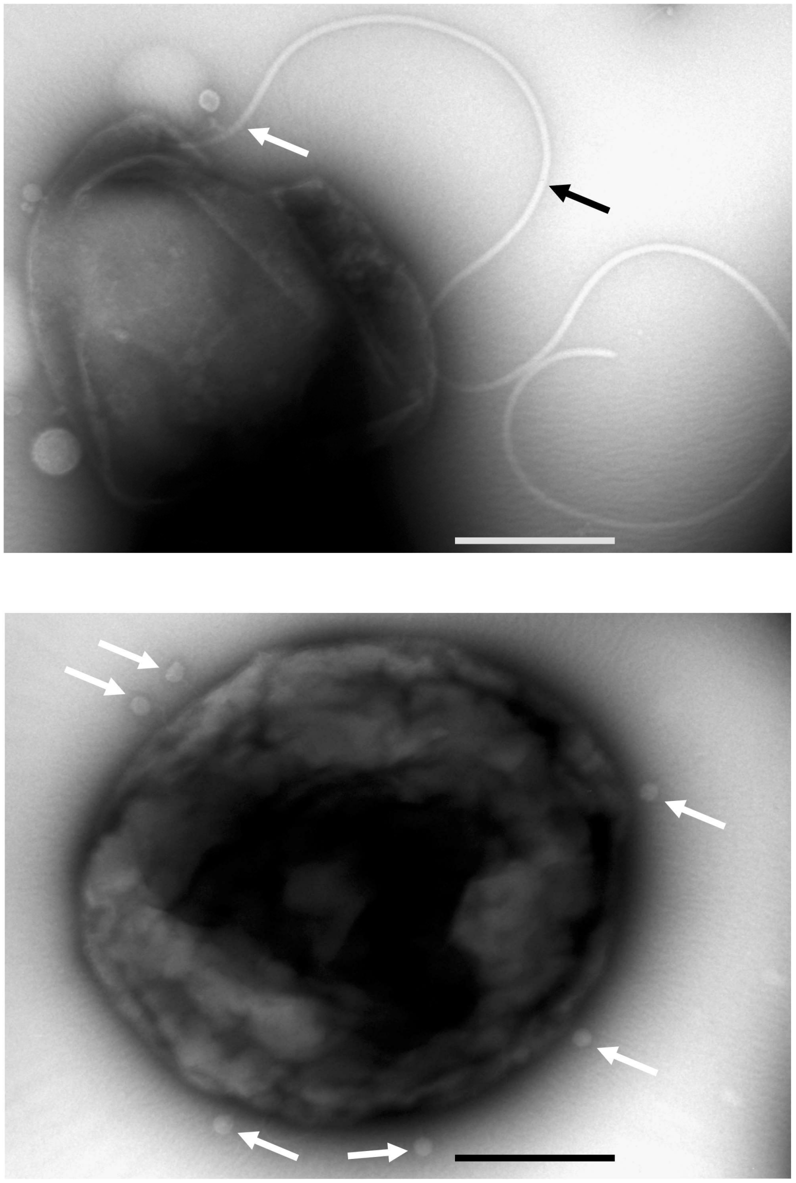

Supplementary Figure S2: Attachment of virus Blf4 to *M. bourgensis* E02.3. Top: virus (white arrow) attachment to cell appendage (presumably flagellum, black arrow) originating from a *M. bourgensis* E02.3 cell. Bottom: several virus particles (white arrows) attached to a *M. bourgensis* E02.3 cell; bars = 500 nm; TEM was conducted as described in Materials and Methods.

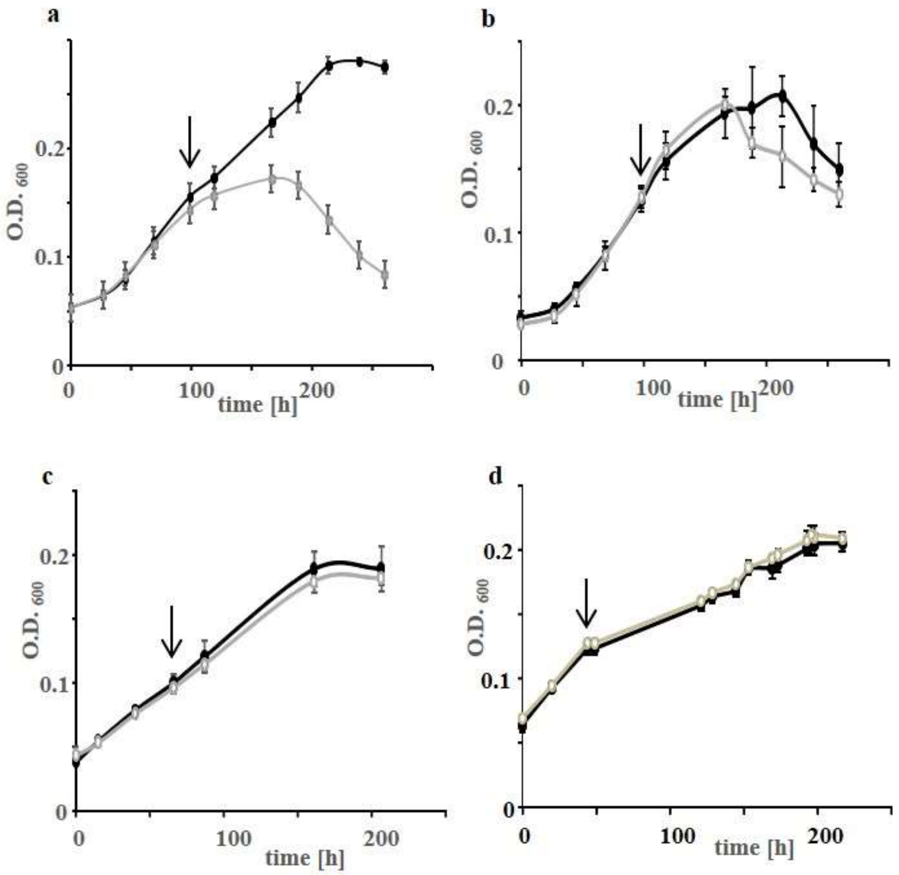

Supplementary Figure S3: Host range of virus Blf4. Representative growth curve of different *Methanoculleus* strains (50 ml, black lines), (a) *M. bourgensis* E02.3, (b) *M. bourgensis* MS2^T (DSM 3045), (c) *M. marisnigri* AN8 (DSM 4552), and (d) *M. thermophilus* CR1^T (DSM 2373). Strains (grey lines) were challenged with 0.5 ml filtrated Blf4-lysed culture supernatant (indicated with arrow) and turbidity was measured at 600 nm; shown are average values and their standard deviations (error bars) of three biological replicates.

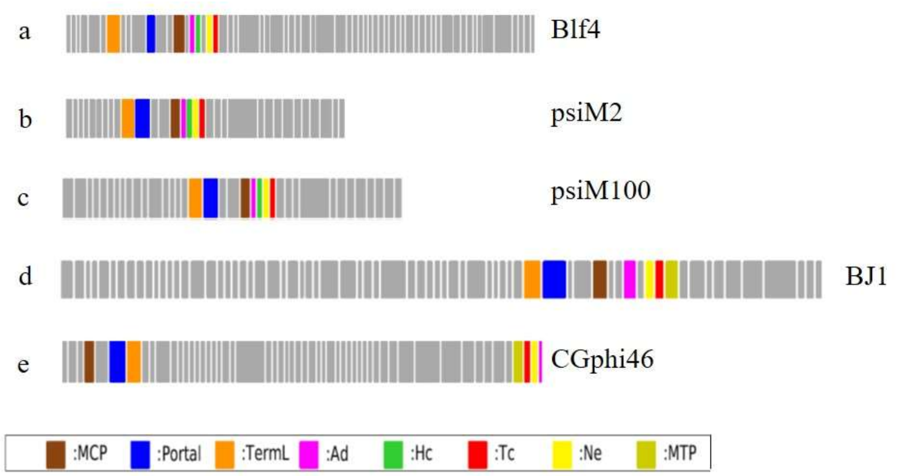

Supplementary Figure S4: VIRFAM analysis by head-neck-tail module search

The genomes of virus Blf4 (a) and the four members of the *Siphoviridae* infecting archaea [virus psi(Ψ)M2 (b); psi(Ψ)M100 (c); BJ1 (d) and CGphi46 (e)] were analyzed using the head-neck-tail module of the VIRFAM analysis tool (see Materials and Methods). Highlighted are the genes for major capsid protein (MCP, brown), the portal protein (Portal, blue), terminase (TermL, orange), head tail adapter (Ad; pink), head closure protein (Hc, green), tail completion protein (Tc. red), neck protein (Ne, yellow), and major tail protein (MTP, beige).
